# Transmembrane helices mediate the formation of a stable ternary complex of b_5_R, cyt b_5_, and SCD1

**DOI:** 10.1038/s42003-022-03882-z

**Published:** 2022-09-12

**Authors:** Jiemin Shen, Gang Wu, Ah-Lim Tsai, Ming Zhou

**Affiliations:** 1 Verna and Marrs McLean Department of Biochemistry and Molecular Biology, Baylor College of Medicine, Houston, TX 77030 USA; 2 Division of Hematology-Oncology, University of Texas McGovern Medical School, Houston, TX 77030 USA

**Keywords:** Enzymes, Enzymes

## Abstract

Mammalian cytochrome b_5_ (cyt b_5_) and cytochrome b_5_ reductase (b_5_R) are electron carrier proteins for membrane-embedded oxidoreductases. Both b_5_R and cyt b_5_ have a cytosolic domain and a single transmembrane (TM) helix. The cytosolic domains of b_5_R and cyt b_5_ contain cofactors required for electron transfer, but it is not clear if the TM helix has function beyond being an anchor to the membrane. Here we show that b_5_R and cyt b_5_ form a stable binary complex, and so do cyt b_5_ and stearoyl-CoA desaturase-1 (SCD1). We also show that b_5_R, cyt b_5_ and SCD1 form a stable ternary complex. We demonstrate that the TM helices are required for the assembly of stable binary and ternary complexes where electron transfer rates are greatly enhanced. These results reveal a role of the TM helix in cyt b_5_ and b_5_R, and suggest that an electron transport chain composed of a stable ternary complex may be a general feature in membrane-embedded oxidoreductases that require cyt b_5_ and b_5_R.

## Abstract (web summary)

The transmembrane domains of mammalian cytochrome b_5_ (cyt b_5_), cyt b_5_ reductase (b_5_R), and stearoyl-CoA desaturase-1 (SCD1) form stable binary complexes between cyt b_5_/b_5_R or cyt b_5_/SCD1 and a ternary complex, which enhance electron transfer rates.

## Introduction

Cytochrome b_5_ (cyt b_5_) and cytochrome b_5_ reductase (b_5_R) are obligatory partners for a number of membrane-embedded oxidoreductases such as fatty acid desaturases and elongases, oxygenases, and cytochrome P450s (cyt P450)^1,2^. b_5_R and cyt b_5_ form part of an electron transport chain that transfers electrons from a reductant, nicotinamide dinucleotide (NADH) or nicotinamide dinucleotide phosphate (NADPH), first to the flavin adenine dinucleotide (FAD) cofactor of b_5_R^3^, then the heme moiety of cyt b_5_^4^ and finally the metal ions in the catalytic center of the membrane-embedded oxidoreductases.

**Fig. 1:**
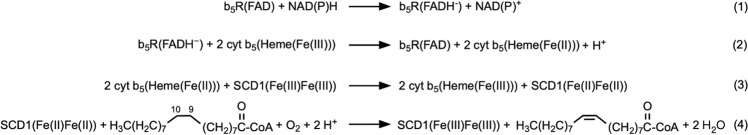
Stepwise reactions in the electron transport chain of b_5_R, cyt b_5_, and SCD1. The redox states of cofactors in proteins are indicated in the parentheses.

Mammalian stearoyl CoA desaturase-1 (SCD1) is a membrane-embedded oxidoreductase that catalyzes the formation of the first double-bond in saturated fatty acids. SCD1 has a major role in the regulation of fatty acid metabolism and membrane synthesis and is a validated drug target for many types of cancers^5–8^, neurodegenerative diseases^9–11^, and metabolic diseases^12–15^. The double-bond formation is catalyzed by a diiron center in SCD1, which requires electrons from NAD(P)H delivered by b_5_R and cyt b_5_ (Fig. 1).

Extensive structural and functional studies have been conducted on the soluble forms of b_5_R and cyt b_5_ that lack their transmembrane (TM) domains. These studies show that the soluble domains of b_5_R and cyt b_5_ are sufficient to support electron transfer, and that charged residues on the surface of the soluble domains mediate their interactions^16–21^. However, less attention has been paid to the role of their TM domains, and a stable cyt b_5_-b_5_R binary complex has not been isolated.

Studies of SCD1 have shown that b_5_R and cyt b_5_ are required for the activity of SCD1^12,22–24^. Whether cyt b_5_ and SCD1 form a stable complex via interactions in their TM domains and whether such a stable complex enhances the activity of SCD1 have not been explored. These questions are important in terms of understanding how each redox component of the electron transport chain function in the native environment and the mechanisms of electron transfer. Knowledge on stable binary or ternary complexes of electron transfer partners is relevant in developing novel strategies to inhibit SCD1 or other membrane-bound oxidoreductases.

## Results

### Colocalization and formation of stable binary and ternary complexes in cells

**Fig. 2:**
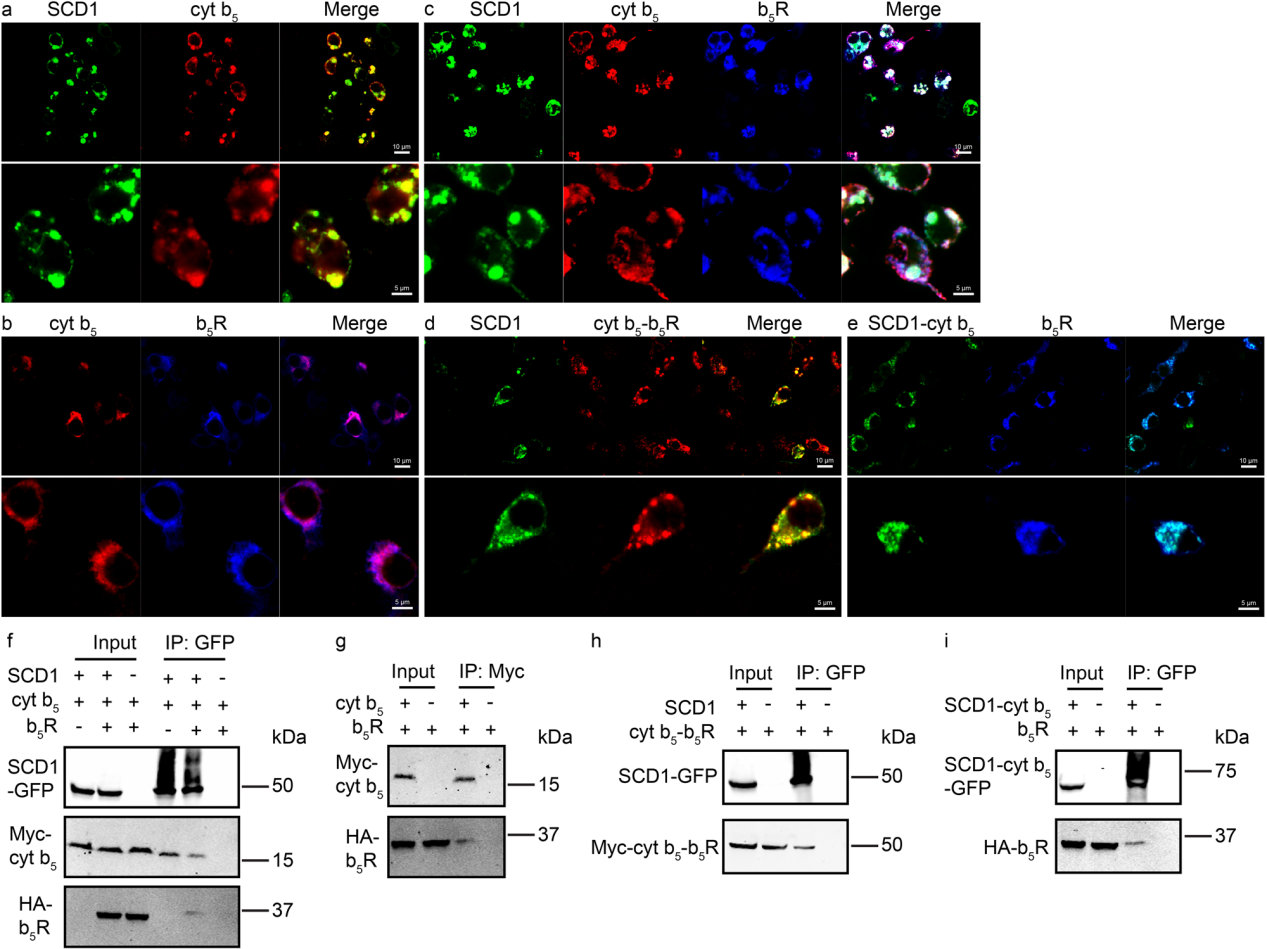
Colocalization and co-immunoprecipitation (co-IP) of SCD1, cyt b5, b5R, and their binary fusions. Confocal microscopy images show the subcellular distribution and colocalization of **a** SCD1-GFP (green) and Myc-cyt b_5_ (red); **b** Myc-cyt b_5_ (red) and HA-b_5_R (blue); **c** SCD1-GFP (green), Myc-cyt b_5_ (red), and HA-b_5_R (blue); **d** SCD1-GFP (green) and Myc-cyt b_5_-b_5_R (red); **e** SCD1-cyt b_5_-GFP (green) and HA-b_5_R (blue). **a**–**e** Images in the top and bottom panels are from the same samples of different magnifications. White scale bars represent 10 µm for the top panels and 5 µm for the bottom panels. **f** Co-IP of SCD1, cyt b_5_, and b_5_R. Cells co-expressing SCD1-GFP and Myc-cyt b_5_ (left lane) were solubilized, and the lysate was immunoprecipitated with GFP nanobody-conjugated resins. Detection of Myc-cyt b_5_ after extensive wash of the resins indicates some stable complex assembly between SCD1 and cyt b_5_. Similarly, ternary complex formation was demonstrated from cells co-expressing tagged SCD1, cyt b_5_, and b_5_R (middle lanes). The lysate from cells expressing tagged cyt b_5_ and b_5_R (right lane) served as a negative control to exclude the possibility of non-specific binding of cyt b_5_ and b_5_R to resins and non-specificity of antibodies used in western blots. **g** Co-IP of cyt b_5_ and b_5_R shows the existence of some stable cyt b_5_-b_5_R complex. Unlike in **f**, lysates were immunoprecipitated with anti-Myc antibodies and protein A resins to capture Myc-cyt b_5_. **h** Co-IP of SCD1 with the binary fusion of cyt b_5_-b_5_R. **i** Co-IP of the binary fusion of SCD1-cyt b_5_ with b_5_R. For all the input lanes, 6% of cell lysate was loaded. IP, immunoprecipitation. All the data are from one representative experiment of at least two independent repeats.

We first examined the colocalization of SCD1, cyt b_5,_ and b_5_R in cells. We fused mouse SCD1 with a green fluorescent protein (GFP), mouse cyt b_5_ with a Myc tag, and mouse b_5_R with a hemagglutinin (HA) tag, and monitored the cellular localization of the three proteins by immunofluorescence confocal microscopy. When SCD1 was expressed, a meshwork-like distribution of GFP fluorescence was observed (Supplementary Fig. 1a–f), consistent with its localization to the endoplasmic reticulum (ER) membranes^25^. Cells co-expressing SCD1 and cyt b_5_ exhibited overlapping fluorescence, and although the fluorescence from cyt b_5_ clustered with most of that from SCD1 as shown in yellow in the merged image (Fig. 2a), cyt b_5_ seemed to have a wider distribution than SCD1 likely due to different expression levels of these proteins and participation of cyt b_5_ in multiple redox pathways. Colocalization was also evident in cells co-expressing cyt b_5_ and b_5_R (Fig. 2b), consistent with their roles in mediating electron transfer to redox enzymes. Co-localization of all three proteins was observed when the three were co-expressed in the same cells (Fig. 2c). These observations led us to further examine if cyt b_5_ form stable binary complexes with b_5_R or SCD1 and if the three form a stable ternary complex.

To test whether the proximity in their expression patterns leads to the formation of stable binary or ternary complexes, we next examined their interactions by co-immunoprecipitation (co-IP). We found that cyt b_5_ co-immunoprecipitates with SCD1, b_5_R with cyt b_5_, and b_5_R and cyt b_5_ with SCD1 (Fig. 2f–g). We also generated binary fusions by connecting SCD1 and cyt b_5_, and cyt b_5_ and b_5_R to test their assembly with b_5_R or SCD1, respectively. The SCD1-cyt b_5_ fusion protein has a C-terminal GFP tag, and cyt b_5_-b_5_R fusion protein has an N-terminal Myc tag. Colocalization analysis showed that SCD1 and cyt b_5_-b_5_R (Fig. 2d), SCD1-cyt b_5_ and b_5_R (Fig. 2e) are in close proximity in cells. Co-IP results indicate that the binary fusions led to higher level of the ternary complex than co-expression of SCD1, cyt b_5,_ and b_5_R (Fig. 2h–i).

### Stable binary complex between b_5_R and cyt b_5_

**Fig. 3:**
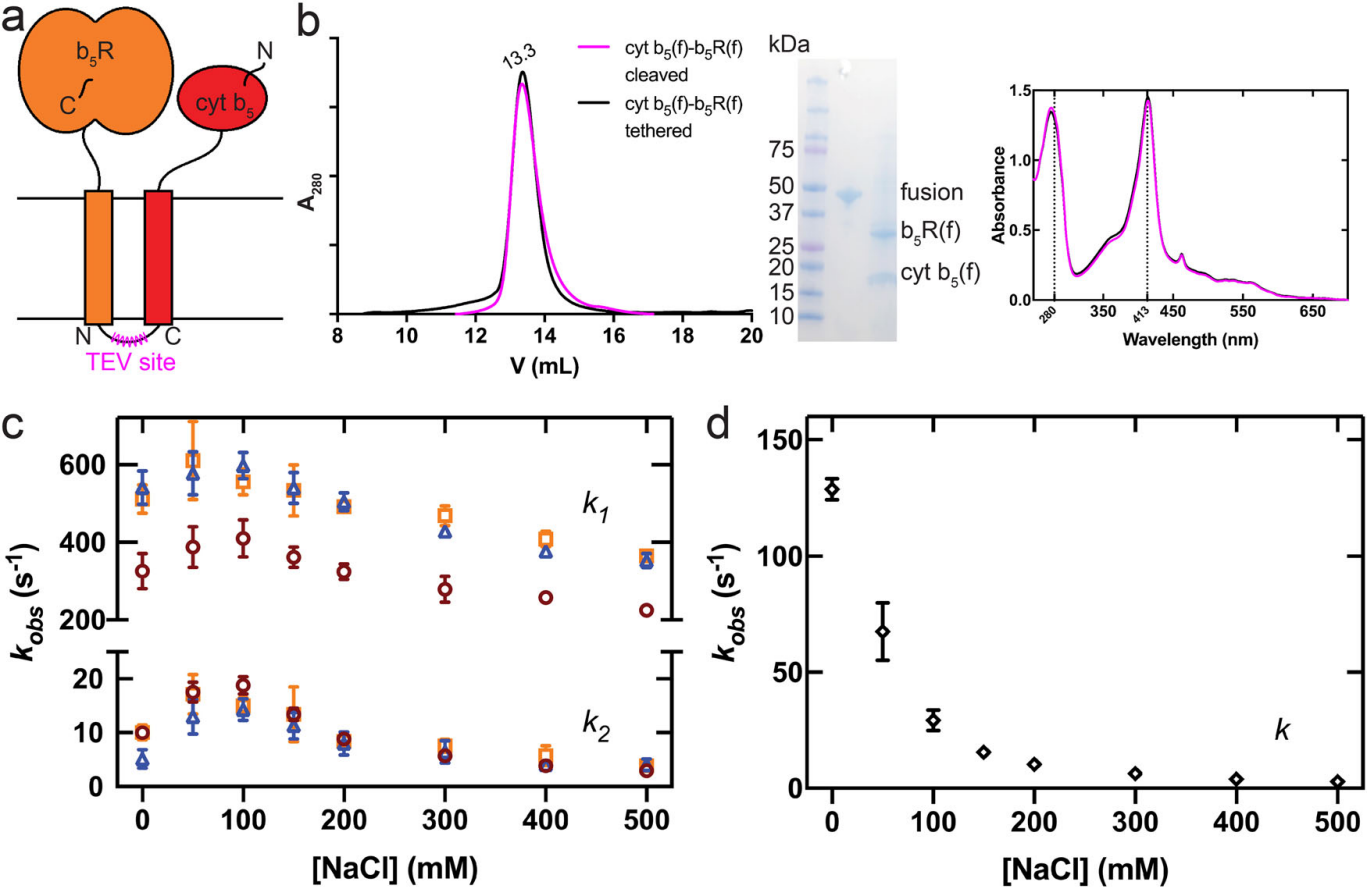
Full-length cyt b5 and full-length b5R can form a stable complex with faster electron transfer kinetics. **a** Schematic diagram of cyt b_5_-b_5_R fusion constructs. The N and C denote the N-terminus and C-terminus of cyt b_5_ and b_5_R. The magenta zigzag line indicates the placement of a TEV protease site on the linker region connecting the cyt b_5_ and b_5_R. **b** Size-exclusion chromatography (SEC) profile (left), SDS-PAGEl image (inset), and UV-Vis spectra (right) of the linker-cleaved (magenta) and tethered (black) fusion of SCD1-cyt b_5_. Almost identical SEC profiles and optical spectra suggest the stable assembly between cyt b_5_ and b_5_R. **c** Ionic strength-dependent electron transfer in different constructs of cyt b_5_–b_5_R: linker-cleaved full-length (orange); tethered full-length (blue); tethered soluble without TM domains (red). The upper and lower half of the plot represent the rate constants of the fast phase (*k*_*1*_) and the slow phase (*k*_*2*_), respectively. **d** Ionic strength-dependent electron-transfer between individual b_5_R and cyt b_5_. Different from those of cyt b_5_-b_5_R fusions, the time courses with individual b_5_R and cyt b_5_ are monophasic. Error bars represent standard error of the mean (SEM) from three independent repeats (*n* = 3).

We proceeded to the large-scale production of cyt b_5_ and b_5_R complex for further biochemical characterizations. However, simply co-expressing the full-length cyt b_5_ and b_5_R did not produce sufficient amount of complex. To increase the yield of the stable complex, and encouraged by previous reports on the production of stable dimeric membrane proteins after fusing two monomers^26–28^, we adopted the strategy of expressing a fusion protein of full-length cyt b_5_ and b_5_R as a concatenated chimera with a linker connecting the C-terminus of cyt b_5_ to the N-terminus of b_5_R (Fig. 3a). The linker contained a tobacco etch virus (TEV) protease recognition site and can be cleaved after purification. The fusion protein was expressed and purified, and the yield was sufficient for further biochemical studies (Fig. 3b). The fusion protein contains a heme, as indicated by the UV/Vis absorption spectrum (Fig. 3b), and elutes as a single peak on a size-exclusion chromatography (SEC) column. After the linker was cleaved by the TEV protease, cyt b_5_ and b_5_R stayed together as a stable complex as indicated by the same elution volume as that of the fusion protein (Fig. 3b). However, when the soluble domains of cyt b_5_ and b_5_R were expressed as a fusion protein, the two soluble domains did not stay as a stable complex after cleavage of the linker (Supplementary Fig. 3a, d). These results indicate that interactions between the TM domains are required to maintain the stable binary complex.

We next measured the rate of electron transfer in the stable binary complex of cyt b_5_ and b_5_R. We measured the reduction of cyt b_5_ in the context of a stable complex with b_5_R or as an individual protein mixed with b_5_R. We found that the time course of cyt b_5_ reduction can be fit with a biphasic exponential function (*k*_*1*_ and *k*_*2*_) when cyt b_5_ and b_5_R were in a stable binary complex (Supplementary Fig. 2a). The biphasic kinetics of electron transfer was also observed in the soluble fusion of cyt b_5_–b_5_R with similar rates to those of the full-length fusion. In contrast, the time course can be fitted with a single exponential function when the soluble forms of individual cyt b_5_ and b_5_R were mixed (Supplementary Fig. 2b). The electron transfer rate (*k*_*1*_) is ~34-fold faster in the stable binary complex than that in the mixture of individual proteins in 150 mM NaCl. Thus, the formation of a stable cyt b_5_–b_5_R complex enhances the spatial proximity and the precise alignment of the two soluble domains to facilitate electron transport.

The rate of electron transfer between soluble forms of b_5_R and cyt b_5_ is known to be sensitive to ionic strength^29^. We next tested how the ionic strength affects the rate of electron transfer from b_5_R to cyt b_5_ in the stable complex. The time courses of cyt b_5_ reduction in the stable binary complex and the mixture of two individual proteins were followed in buffer with different NaCl concentrations, and the rates were calculated from either double exponential fitting for the fusion proteins or single exponential fitting for the mixture of individual proteins. The full-length and soluble fusions displayed a similar trend of ionic strength dependence where the electron transfer rates (*k*_*1*_ and *k*_*2*_) peaked at ~50 mM NaCl and decreased as [NaCl] increased (Fig. 3c). When the two proteins form a stable binary complex, the difference between no NaCl and 150 mM NaCl is only 1.04-fold. However, when the two proteins were not assembled as a stable complex, ionic strength has a significant effect on the rate of electron transfer (Fig. 3d): an ~8-fold decrease was observed from no NaCl to 150 mM NaCl. These results suggest that the formation of a stable binary complex aligns the soluble domains in position for electron transfer so that electrostatic interactions have a much smaller role in guiding and facilitating the proper interactions of the soluble domains.

### Stable binary complex between cyt b_5_ and SCD1

**Fig. 4:**
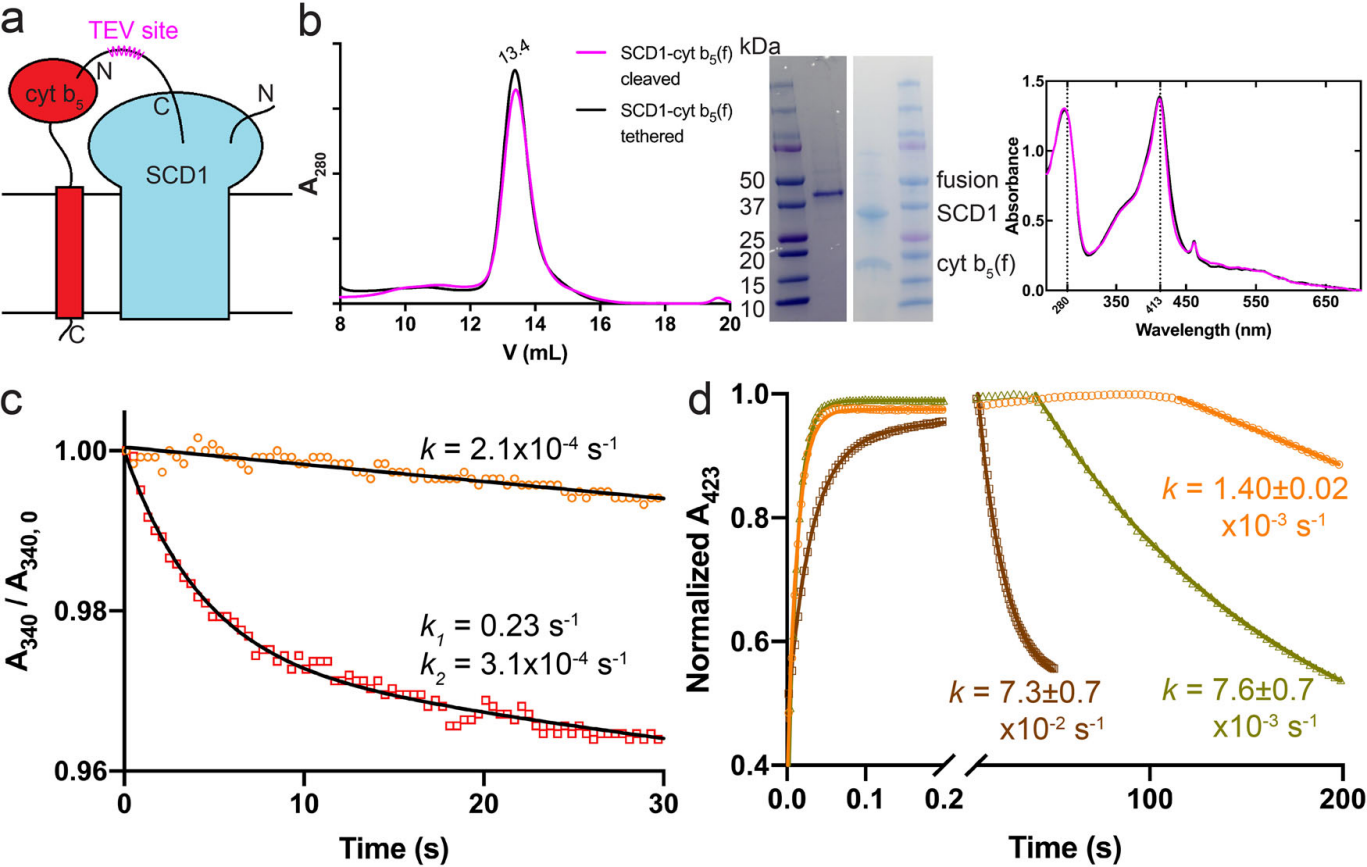
SCD1 and full-length cyt b5 can form a stable complex with faster electron transfer kinetics. **a** Schematic diagram of SCD1-cyt b_5_ fusion constructs. **b** SEC profile (left), SDS-PAGE image (inset), and UV-Vis spectra (right) of the linker-cleaved (magenta) and tethered (black) fusion of SCD1-cyt b_5_. Almost identical SEC profile and optical spectra suggest the stable assembly between SCD1 and cyt b_5_. **c** Biphasic kinetics of NADH consumption by b_5_R with SCD1-cyt b_5_ fusion in the presence of substrate stearoyl-CoA (red) compared to the slow linear decrease of NADH in the absence of substrate (orange). The absorbance of NADH at 340 nm (A_340_) was measured and the *y* axis is the normalized A_340_ against the initial values (A_340, 0_). Excess NADH was used in the measurements. **d** The accelerated electron transfer between reduced cyt b_5_ and SCD1 in the SCD1-cyt b_5_ complex (brown) compared to that between the individual cyt b_5_ and SCD1 (yellow) and auto-oxidation of cyt b_5_ (orange). The Soret absorbance of reduced cyt b_5_ at 423 nm was monitored. One molar equivalent of NADH was added, which resulted in the initial rising phases of the fast electron transfer to cyt b_5_ via b_5_R. Rate constants (*k*) are denoted as mean ± SEM calculated from three independent repeats (*n* = 3).

We next investigated whether the full-length cyt b_5_ forms a stable complex with SCD1. We found that co-expression of the two proteins does not produce a high level of stable SCD1-cyt b_5_ binary complex. We then applied the fusion protein strategy and linked the C-terminus of SCD1 to the N-terminus of the full-length cyt b_5_ with a TEV recognition site in the linker (Fig. 4a). The SCD1-cyt b_5_ fusion protein had a sufficient yield and eluted as a single peak on a SEC column (Fig. 4b). After cleavage of the linker, SCD1 and cyt b_5_ stayed together as a stable complex as indicated by the single peak on a SEC column; the UV-Vis spectra of the elution peaks are identical before and after TEV protease treatment (Fig. 4b). When the soluble domain of cyt b_5_ was fused to SCD1, the two did not stay together as a stable complex after the linker was cleaved, as indicated by two peaks on a SEC column (Supplementary Fig. 3b, e), indicating that the TM domain of cyt b_5_ is required for the formation of the stable binary complex.

We then examined if the stable binary complex of SCD1-cyt b_5_ is capable of receiving electrons from b_5_R, and transferring electrons from cyt b_5_ to SCD1. We first measured the enzymatic activity of SCD1-cyt b_5_ by including b_5_R and NADH, and we monitored the reaction by following the consumption of NADH in the presence and absence of a substrate stearoyl-CoA. As shown in Fig. 4c, the fast-phase rate of NADH consumption is 1000-fold faster than the rate in the absence of stearoyl-CoA. Next, we measured individual electron transfer steps by following the optical change of cyt b_5_. The reduced form of cyt b_5_ was monitored by its Soret peak at 423 nm. Under the anaerobic condition, cyt b_5_ in the stable binary complex of SCD1-cyt b_5_ was first reduced by b_5_R after equimolar of NADH was added, corresponding to the fast-rising phase (Fig. 4d, *t* < 20 s). After the exhaustion of NADH, the phase of re-oxidation (*t* > 20 s) of cyt b_5_ by SCD1 appeared. The electron transfer rate between cyt b_5_ and SCD1 in the stable binary complex is 7.3 ± 0.7 × 10^−2^ s^−1^, almost 10-fold higher than that in the mixture of individual cyt b_5_ and SCD1 (Fig. 4d). These results indicate that the stable binary complex of SCD1 and cyt b_5_ is fully functional, and that the formation of the stable binary complex likely facilitates the alignment and interactions of the two proteins inductive to electron transfer.

### Stable ternary complex between b_5_R, cyt b_5_, and SCD1

**Fig. 5:**
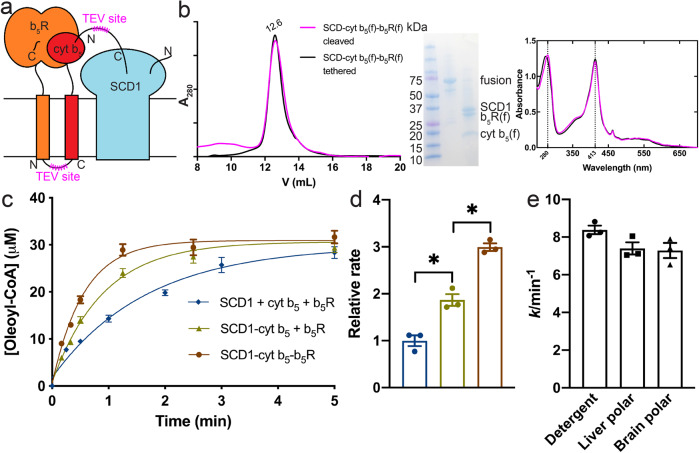
SCD1, full-length cyt b5, and full-length b5R can form a stable complex with faster electron transfer kinetics. **a** Schematic diagram of SCD1-cyt b_5_ fusion constructs. **b** SEC profile (left), SDS-PAGE image (inset), and UV-Vis spectra (right) of the linker-cleaved (magenta) and tethered (black) fusion of SCD1-cyt b_5_-b_5_R. Almost identical SEC profile and optical spectra suggest the stable assembly among SCD1, cyt b_5_, and b_5_R. **c** Time courses of oleoyl-CoA production (*n* = 3) in: individual SCD1, cyt b_5_ and b_5_R (blue); the binary complex of SCD1-cyt b_5_ and individual b_5_R (yellow); the ternary complex of SCD1-cyt b_5_-b_5_R (brown). **d** Initial rate comparison among conditions in **c** (*n* = 3). Asterisks indicate significant different (*p* < 0.05) in pairwise *t* tests. **e** Initial rates of the SCD1-cyt b_5_-b_5_R complex in detergent or liposomes. Error bars represent SEM from three independent repeats (*n* = 3).

Encouraged by the biochemical isolation of the two stable binary complexes, we attempted the production of a stable ternary complex of SCD1, cyt b_5_ and b_5_R. We took a similar strategy of connecting all three full-length proteins with TEV protease-cleavable linkers as shown in Fig. 5a. The purified fusion protein eluted as a single peak from a SEC column (Fig. 5b). After the linkers were cleaved, all three proteins stayed together as a stable ternary complex (Fig. 5b). Similarly with results from the binary complexes, SCD1 does not stay together with soluble cyt b_5_ and b_5_R in SEC (Supplementary Fig. 3c, f).

The stable ternary complex is fully functional, as indicated by the production of oleoyl-CoA when supplied with NADH and stearoyl-CoA (Fig. 5c). The rate of oleoyl-CoA production is significantly faster than those in individual proteins or the stable SCD1-cyt b_5_ binary complex (Fig. 5d), indicating that the stable ternary complex forms an efficient electron transport chain and enhances the alignment of electron donors and acceptors. When the stable ternary complex is reconstituted into liposomes composed of lipids from liver polar extract or brain polar extract, it maintains the desaturation activity (Fig. 5e).

### Mutational studies of binary and ternary complexes

**Fig. 6:**
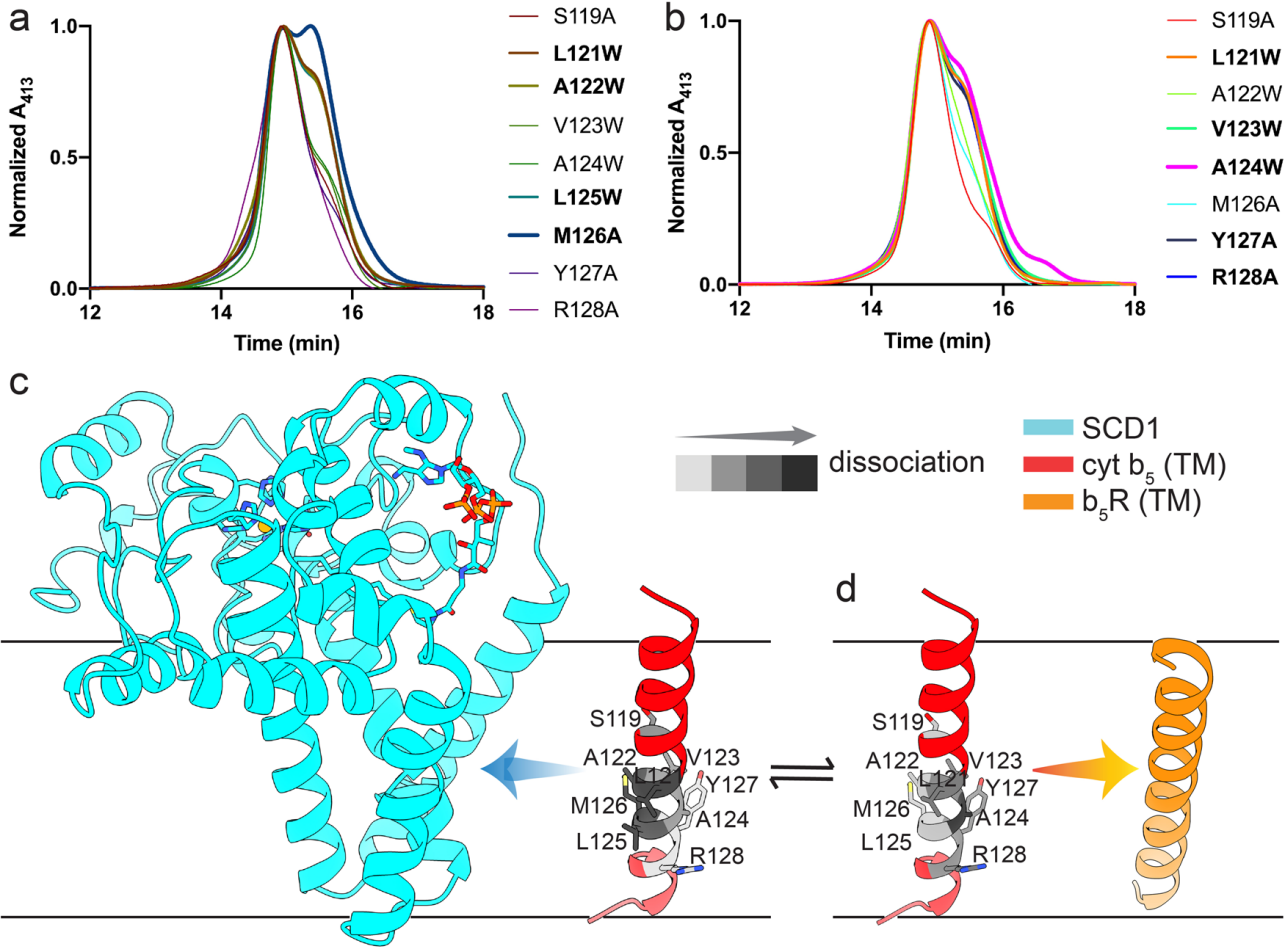
Mutations on the TM domains of SCD1, cyt b5, and b5R partially disrupt the stable complex assembly of the SCD1-cyt b5 and the cyt b5-b5R. SEC profiles of: **a** linker-cleaved SCD1-cyt b_5_ complexes, and **b** linker-cleaved cyt b_5_-b_5_R complexes with mutations on TM domains. Residues on the TM helix of cyt b_5_ were mutated to either an Ala from a polar or large hydrophobic residue or a bulky Trp from a small hydrophobic residue. The absorbance at 413 nm from the heme group in cyt b_5_ was monitored. Mutants causing highest complex dissociation were highlighted. The model of complex assembly in: **c** SCD1 (cyan) and TM helix of cyt b_5_ (red); and **d** TM helix of cyt b_5_ (red) and b_5_R (orange). Residues important for complex assembly are shown as sidechain sticks and are colored per their degree of causing complex dissociation when mutated based on results in **a**, **b**. Darker color represents a larger effect on disrupting complex assembly. One-sided arrows indicate the side of the TM helix of cyt b_5_ participating in the binary complex formation.

Attempts to determine the structures of stable binary complexes of cyt b_5_ and SCD1, and cyt b_5_ and b_5_R, or the stable ternary complex of cyt b_5_, b_5_R and SCD1 by either X-ray crystallography or cryo-electron microscopy have not been successful. To gain further insight into the interactions between the TM domains, we made mutations to the TM region of cyt b_5_ and assessed the effect on the formation of the two stable binary complexes. We used the I-TASSER^30^ and AlphaFold2^31^ programs to generate a structural model of the TM helix of cyt b_5_ and b_5_R (Methods). We made single-point mutations to 10 consecutive residues, 119 – 128, of the predicted TM helix of cyt b_5_ in the context of either the SCD1-cyt b_5_-TEV chimera or cyt b_5_-b_5_R-TEV chimera. Small hydrophobic residues were replaced with a bulky Trp, and polar or large hydrophobic residues were replaced with Ala. Tryptophan substitution on TM domains was shown to weaken or disrupt dimerization of the ClC transporters^32,33^, although due to the amphipathic nature of its side chain, it may also shift the position of the TM helix. All but one mutants (residue 120) expressed, which allowed for a systematic perturbation of the stable binary complexes. After the fusion proteins were purified and cleaved by TEV protease, we examined the binary complexes by SEC. By monitoring the absorbance at 413 nm, where the binary complexes and monomeric cyt b_5_ have the same molar extinction coefficient, we were able to estimate the ratio of the heterodimer to monomer (Fig. 6 and Supplementary Fig. 4a, b).

In the SCD1-cyt b_5_ complex, the strongest effect was observed on M126A (~50% dissociation), with L121W, A122W, and L125W have a lower effect (~40% dissociation), and Y127A, R128A have the lowest effect (~10% dissociation) (Fig. 6a). In the cyt b_5_-b_5_R complex, A124W and R128A have the largest effect (~40% dissociation), with L121W and V123W, Y127A follows, and S119A, A122W, and M126A have the lowest effect (Fig. 6b). Overall, the effect of point mutations on the cyt b_5_-b_5_R complex tend to be more modest compared to these on the SCD1-cyt b_5_ complex. The varying degrees of complex dissociation in mutants were mapped onto the TM model of cyt b_5_ as shown in Fig. 6c, d. It seems that cyt b_5_ engages SCD1 or b_5_R using the opposite sides of its TM helix: the side of M126 interacts with SCD1, while the side of A124 and R128 with b_5_R (Fig. 6c, d). These results are consistent with the conclusion that the TM domains mediate the formation of stable binary and ternary complexes of SCD1, cyt b_5_, and b_5_R.

**Fig. 7:**
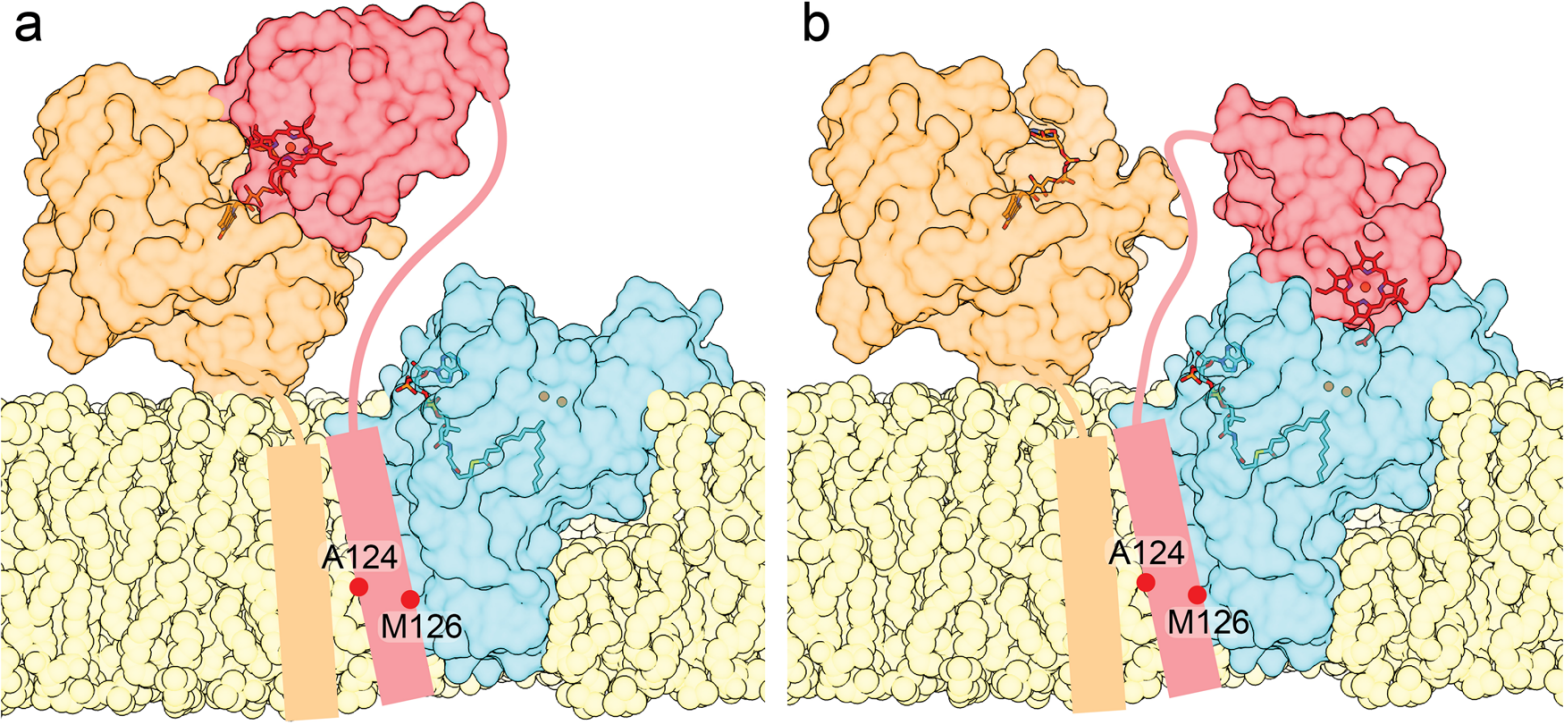
Model of the stable SCD1-cyt b5-b5R complex. SCD1 (cyan), cyt b_5_ (red), and b_5_R (orange) are shown as translucent surfaces highlighting the diiron center (orange sphere) and acyl-CoA (cyan stick) in SCD1, heme (deep red stick) in cyt b_5_, and FAD (brown stick) in b_5_R. SCD1 forms a complex with the TM helix of cyt b_5_ and b_5_R in lipid bilayer (yellow). The relative positions of two residues on the TM helix of cyt b_5_ are marked. A flexible linker connecting the soluble domain of cyt b_5_ and b_5_R to their TM helix allows the transition of two states: **a** cyt b_5_ receiving an electron from b_5_R; and **b** cyt b_5_ delivering an electron to SCD1.

These results led us to propose a working model of an electron transport chain formed by a stable ternary complex of b_5_R, cyt b_5_, and SCD1 (Fig. 7). Cyt b_5_ and b_5_R form a stable binary complex that further interacts with SCD1 or other membrane-bound oxidoreductases to form a stable ternary complex. While the single TM helix of cyt b_5_ is sandwiched and thus immobilized between b_5_R and SCD1 in the stable ternary complex, its soluble domain is mobile and can alternate between interacting with b_5_R or SCD1 to relay electrons.

Soluble domains of SCD1, cyt b_5_, and b_5_R have clear complementary electrostatic charge distributions (Supplementary Fig. 5a). Soluble domains of SCD1 and b_5_R have positively charged surfaces while that of cyt b_5_ has a negatively charged surface. Although in the context of a stable complex, electrostatic interactions no longer play a major role in the rate of electron transfer (Fig. 2c, d), charge-charge interactions could steer and align the donor-acceptor redox centers in the soluble domains. As a test of electrostatic interactions of the soluble domain of cyt b_5_ with either b_5_R or SCD1, we mutated two charged residues, E43 and E49, on the surface of cyt b_5_ (Supplementary Fig. 5b, c) and measured their binding affinities to either b_5_R or SCD1. The affinities to both b_5_R and SCD1 decreased modestly in these mutants (Table 1 and Supplementary Fig. 6), suggesting that these residues are involved in interactions with both b_5_R and SCD1.

**Table 1:** Binding affinities of soluble cyt b_5_ to SCD1 or soluble b_5_R.

*K*_D_ (µM)	b_5_R	SCD1
cyt b_5_ (WT)	2.71 ± 0.33	4.42 ± 1.77
cyt b_5_ (E43A)	9.63 ± 0.97	11.2 ± 2.6
cyt b_5_ (E49A)	8.45 ± 1.44	8.23 ± 2.49

*K*_D_ values are denoted as mean ± SEM calculated from three measurements (*n* = 3). Sensorgrams are shown in Supplementary Fig. 6.

## Discussion

In summary, we demonstrated that SCD1, cyt b_5_, and b_5_R form a stable ternary complex and that cyt b_5_ forms a stable binary complex with either b_5_R or SCD1. The stable complexes are mediated by the TMs of the proteins, and the rates of electron transfer are significantly enhanced in the complexes. The facilitation of redox communication in binary and ternary complexes is demonstrated in both multiple and single turnover kinetic measurements for product formation and temporal changes of each redox center. The formation of a stable binary complex of b_5_R and cyt b_5_ suggests it may interact with other downstream proteins as a single unit.

Cyt b_5_ is known to have a number of downstream partners, such as fatty acid desaturases and elongases, oxygenases, and cyt P450^1,2^, and this indicates that the soluble domain of cyt b_5_ is capable of interacting with different redox partners. Our study demonstrates that the TM domain of cyt b_5_ interacts with both the upstream partner (b_5_R) and at least one downstream partner, SCD1, to form a stable ternary complex. We speculate that the TM of cyt b_5_ mediate the formation of stable complexes with other downstream enzymes.

Recent studies^34–39^ using nuclear magnetic resonance and molecular dynamic simulation have shown interactions between TM helix of cyt b_5_ and that of cyt P450, an oxidoreductase with a single TM helix. The authors found that when both full-length cyt b_5_ and cyt P450 were incorporated into a single lipid nanodisc^34^, their TMs could interact and that the interactions facilitate electron transport between the soluble domains of cyt b_5_ and cyt P450^37^. Interestingly, a conserved motif on cyt b_5_ (L121–L125) that was thought to interact with cyt P450 in nanodiscs^39^ is also identified in our study to mediate interactions with SCD1 and b_5_R. Such conserved motif may serve to form hydrophobic packings with TM domains of various partners of cyt b_5_.

Understanding the interactions of SCD1, cyt b_5_, and b_5_R and ultimately obtaining structures of the binary and ternary complexes in different redox states will help our understanding of SCD1 and other membrane-embedded oxidoreductases that rely on cyt b_5_ and b_5_R. These interactions could be targeted by reagents that modulate electron transfers and enzymatic activities, and the biochemically stable complexes developed in this study could be used in high-throughput screens to identify such reagents.

## Methods

### DNA constructs

The cDNAs of mouse SCD1 (UniProt ID: P13516), full-length cyt b_5_ (UniProt ID: P56395), and full-length b_5_R (UniProt ID: Q9DCN2) were codon optimized and synthesized. Fusions of SCD1-cyt b_5_, cyt b_5_-b_5_R, SCD1-cyt b_5_-b_5_R were generated by PCR. The linkers between each domain were either a TEV protease site (ENLYFQ/G) for the cleavable fusions or a flexible linker (GGSGGGSG) for the non-cleavable fusions. The SCD1 and SCD1-cyt b_5_ fusions were cloned into a pEG BacMam vector with a TEV protease site prepended to a C-terminal GFP tag. Because the C-terminus of b_5_R ends on the interface of the FAD-binding domain and NADH-binding domain, no extra residue was introduced after the C-terminus of b_5_R domain to preserve its functional integrity. Therefore, SCD1-cyt b_5_-b_5_R was cloned into a pEG BacMam vector with an N-terminal GFP tag. For immunofluorescence imaging and coimmunoprecipitation assays, cyt b_5_ and cyt b_5_-b_5_R with an N-terminal Myc tag, and b_5_R with an N-terminal HA tag were cloned into a pEG BacMam vector. For large-scale protein expression and purification, cyt b_5_, b_5_R, and cyt b_5_-b_5_R were cloned into a pFastBac Dual vector with an octa-histidine tag and a TEV protease site. For Octet binding assays, the soluble domains of cyt b_5_ (4–89) and b_5_R (24–301) were cloned into a pET vector.

### Immunofluorescence imaging

The HEK 293 S cells in *FreeStyle 293* media (Invitrogen/Thermo Fisher) supplemented with 2% fetal bovine serum (FBS; Sigma) were plated one day before transfection onto glass coverslips coated with poly-lysine in a 24-well plate. The cDNAs in pEG BacMam vectors were transfected into cells with Lipofectamine 2000 (Invitrogen/Thermo Fisher) per the manufacturer’s instructions. About 24 h after transfection, cells were fixed with 2% paraformaldehyde for 10 min and then washed three times with phosphate-buffered saline (PBS). Cells were blocked and permeabilized with PBSAT (PBS + 1% bovine serum albumin, BSA + 0.1% Triton X-100) for 10 min. For cells expressing only GFP-tagged proteins, coverslips were washed three times with PBS and mounted onto glass slides with *ProLong* Diamond (Invitrogen/Thermo Fisher). For cells expressing Myc and/or HA tagged proteins, primary antibodies against Myc and/or HA tag diluted in PBSAT (1:200) were added and incubated for 45 min at room temperature (RT). Coverslips were washed three time with PBS before the incubation with Alexa Fluor 555 (for Myc tag) and/or Alexa Fluor 647 (for HA tag) conjugated secondary antibodies (Invitrogen/Thermo Fisher) diluted in PBSAT (1:2000) for 30 min at RT. Finally, coverslips were washed and mounted as mentioned before.

Confocal images were acquired with a Zeiss LSM-710 confocal microscope using a ×63 oil immersion objective (Zeiss, Plan-Apochromat ×63/1.4 Oil DIC M27) with *Immersol* 518 F immersion oil (Zeiss). Alexa Fluor 647, Alexa Fluor 555, and GFP were detected sequentially with 633 nm HeNe laser, 561 nm diode-pumped solid-state laser, and 488 nm Argon laser. Crosstalk between the channels was avoided by adjusting emission regions. Single optical sections at a resolution of 1024 × 1024 pixels were acquired at two different zoom levels (×1.5 and ×4).

### Coimmunoprecipitation

The HEK 293 S cells were plated one day before transfection in a 6-well plate. The pEG BacMam vectors containing target cDNAs were transfected with Lipofectamine 2000 (Invitrogen/Thermo Fisher) and incubated for 2 days. Cells on the plate were washed in PBS before scraping. Cell membranes were solubilized in lysis buffer (20 mM HEPES, pH 7.5, 150 mM NaCl, 10% glycerol) plus 0.2% Triton X-100 and Protease Inhibitor Cocktail (Roche) for 1 h at 4 °C. Cell debris were pelleted by centrifugation. The supernatants of cell lysate were incubated with either pre-equilibrated GFP nanobody-conjugated NHS-Activated Sepharose 4 Fast Flow Agarose (GE Healthcare) or *Pierce* Protein A Agarose (Invitrogen/Thermo Fisher) with rabbit anti-Myc antibodies for 30 min at 4 °C. The resins were extensively washed in lysis buffer plus 0.1% Triton X-100 within 5 min at 4 °C. In all, 4× Laemmli Sample Buffer (Bio-Rad) was added, and samples were run in SDS-PAGE without extra elution steps. Bands of target proteins were visualized by western blotting (Supplementary Fig. 7) with mouse anti-GFP (Invitrogen/Thermo Fisher) (1:1000), anti-Myc (1:500), and anti-HA (1:500) antibodies as primary antibodies and IRDye-800CW anti-mouse IgG (Licor) (1:5000) as the secondary antibody. Images were taken in an Odyssey infrared scanner (Licor).

### Large-scale expression and purification of proteins

Expression of SCD1-containing proteins (SCD1, SCD1-cyt b_5_, and SCD1-cyt b_5_-b_5_R) was conducted in HEK 293 S cells using the BacMam system^40^. Baculoviruses were generated from pEGBacMam vectors with target cDNAs and amplified in Sf9 (*Spodoptera frugiperda*) cells. HEK 293 cells were maintained in *FreeStyle 293* media (Invitrogen/Thermo Fisher) supplemented with 2% FBS (Sigma) in a 37 °C incubator with 8% CO_2_ atmosphere at 100 rpm. Baculoviruses after three passages (P3) were added to HEK 293 S cells at a density of 3 × 10^6^ mL^−1^ at a 7.5% v/v ratio and incubated overnight before adding 10 mM sodium butyrate and lowering the temperature to 30 °C. Media were supplemented with transferrin and ferric chloride^41^. 0.5 mM δ-aminolevulinic acid and 100 µM riboflavin were added in media to enhance the biosynthesis of the heme and FAD group, respectively. Three days after infection, cells were harvested and resuspended in lysis buffer plus Protease Inhibitor Cocktail (Roche), 1 mM phenylmethylsulfonyl fluoride (PMSF), 5 mM MgCl_2_ and DNase I. Solubilization of cell membranes was achieved by incubating with 30 mM n-dodecyl-β-d-Maltopyranoside (DDM, Anatrace) for 2 h at 4 °C under gentle agitation. Insoluble fractions were pelleted by centrifugation at 55,000 × *g* for 40 min. Target proteins in supernatants were captured by GFP nanobody resins during 1 h incubation at 4 °C. After washing the resins with 20 CV washing buffer (lysis buffer plus 1 mM DDM), the GFP-tagged proteins were released by TEV protease digestion during which the TEV protease site linkers in the cleavable fusions were also cleaved. The eluents were concentrated (Amicon 50-kDa cutoff, Millipore) and loaded onto a SEC column (Superdex 200 10/300 GL, GE Health Sciences) equilibrated with FPLC buffer (20 mM HEPES, pH 7.5, 150 mM NaCl, 1 mM DDM).

Expression of full-length cyt b_5_, full-length b_5_R, and cyt b_5_-b_5_R was conducted in High Five (*Trichoplusia ni*) cells using the Bac-to-Bac system. Baculoviruses were generated from pFastBac Dual vectors with target cDNAs. 1.5% v/v of P3 virus was added to cells at a density of 3 × 10^6^ mL^−1^. The δ-aminolevulinic acid (Santa Cruz) and/or riboflavin (Sigma) were supplemented in media as mentioned above. Cells were harvested three days after infection. Membranes were solubilized by 30 mM DDM for 2 h at 4 °C under gentle agitation. Insoluble fractions were pelleted by centrifugation at 55,000 × *g* for 40 min. The His-tagged proteins in supernatants were captured by cobalt-based affinity resins (Talon, Clontech) during 1 h incubation at 4 °C. The resins were washed with 3 × 20 CV washing buffer plus imidazole up to 10 mM. The target proteins were released by TEV protease digestion. The eluents were concentrated (Amicon 50-kDa cutoff, Millipore) and loaded onto a SEC column (Superdex 200 10/300 GL, GE Health Sciences) equilibrated with FPLC buffer.

### UV-Vis spectroscopy and enzymatic assays

UV-Vis spectra were recorded using a Hewlett-Packard 8453 diode-array spectrophotometer (Palo Alto, CA). The time courses of the NADH consumption at 340 nm and the spectral change of cyt b_5_ heme at 423 nm were obtained with an Applied Photophysics (Leatherhead, UK) model SX-18MV stopped-flow instrument. The observed rates, *k*_obs_, were obtained by fitting the time courses to either 1- or 2-exponential functions.

Continuous turnover reactions of SCD1, the binary complex of SCD1-cyt b_5_, and the ternary complex of SCD1-cyt b_5_-b_5_R were performed in FPLC buffer. Briefly, 3 µM of: 1) SCD1 plus an equimolar of cyt b_5_ and b_5_R; 2) SCD1-cyt b_5_ plus an equimolar of b_5_R; and 3) SCD1-cyt b_5_-b_5_R in FPLC buffer were incubated with substrate stearoyl-CoA (Sigma). NADH was added to start the reaction. Aliquots of reaction mixtures were retrieved and quenched at different time points and analyzed in high-performance liquid chromatography (HPLC). The initial rates were calculated by linear fitting of time courses within 1 min after the reaction started.

### Liposome reconstitution and enzymatic assays

Liver polar extract or brain polar extract lipids (Avanti) in chloroform were dried under a stream of Argon and then vacuumed for 1 h. Lipids were hydrated with FPLC buffer to a concentration of 10 mg/mL and sonicated to transparency. After three cycles of freeze-thaw, the empty liposomes were extruded through 400 nm filter membrane (NanoSizerTM Extruder, T&T Scientific Corporation) to homogeneity. Prior to the addition of protein, 0.11% (w/v) Triton X-100 (Sigma) was added to destabilize the liposome. SCD1-cyt b_5_-b_5_R complex was added at a protein to lipid ratio of 1:25 (w/w). Detergent was sequentially removed by a total amount of 240 mg per mL of semi-wet Bio-Beads SM-2 (BioRad).

Prior to enzymatic assays, 300 µM of stearoyl-CoA was added to the proteoliposomes. Unilaminar proteoliposomes were formed after three cycles of freeze-thaw and extrusions through 400 nm filter membranes as mentioned above. The desaturation reaction was triggered by the addition of 1 mM NADH. Reaction mixtures were quenched and analyzed by HPLC as described above.

### Size-exclusion chromatography of mutants

Mutations on the TM domains of SCD1, cyt b_5_, and b_5_R were introduced to the constructs of linker-cleavable fusions of SCD1-cyt b_5_ and cyt b_5_-b_5_R by QuikChange site-directed mutagenesis. All mutations were confirmed by sequencing. Expression and purification were done similarly to the wild-type (WT) fusions. Purified proteins were loaded onto a SEC column (SRE-10C SEC-300, Sepax) in an HPLC system with a diode-array detector (SPD-M20A, Shimadzu). Samples were run in FPLC buffer at a flow rate of 0.75 mL/min and monitored at 423 nm. Elution profiles were normalized to the peak corresponding to either the SCD1-cyt b_5_ or the cyt b_5_-b_5_R complex for comparison.

### Modeling of single TM helices

The TM regions of cyt b_5_ and b_5_R were predicted by TMHMM server^42^ and residue 108–134 of cyt b_5_ and residue 1–28 of b_5_R were used to model a TM helix in I-TASSER server^30^. The helical models with the highest C-score were chosen and energy-minimized in CHARMM-GUI Membrane Builder^43–47^. The were protonated at neutral pH and were embedded in a POPC bilayer.

### Octet biolayer interferometry

Soluble cyt b_5_ and b_5_R were expressed in BL21(DE3) cells with pET vectors containing target cDNA. Mutations in cyt b_5_ were introduced by QuikChange site-directed mutagenesis and were confirmed by sequencing. Expression procedures were adapted from established protocols^48,49^. Purification procedures were similar to those for full-length cyt b_5_ and b_5_R as mentioned above.

Biolayer interferometry (BLI) assays were performed at 30 °C under constant shaking at 1000 rpm using an Octet system (FortéBio). The immobilization of ligand proteins on amine reactive second-generation (AR2G) biosensors (Sartorius) was done following the manufacturer’s instructions. Briefly, biosensor tips were activated in 20 mM 1-ethyl-3-[3-dimethylaminopropyl]carbodiimide hydrochloride (EDC) and 10 mM N-hydroxysulfosuccinimide for 300 s. Then the tips were loaded with soluble cyt b_5_ at a concentration of 5 µg/mL in FPLC buffer for 600 s. The tips were quenched in FPLC buffer plus 1 M ethanolamine for 300 s. The tips with immobilized ligands were equilibrated in FPLC buffer plus 0.1% BSA to reduce non-specific binding. Then, they were transferred to wells of a concentration gradient (5, 2.5, and 1.25 µM) of analysts (soluble b_5_R or SCD1) in buffer B for association and returned to the equilibration wells for dissociation. Binding curves were aligned and corrected with the channel of no analyst protein. The association and disassociation phases were fitted with a 1-exponential function to extract *k*_on_ and *k*_off_ of the binding, which were used to calculate dissociation constant *K*_D_.

### Statistics and reproducibility

No statistical method was used to determine sample size. Data used for statistical analyses were from at least three biological repeats. Statical significances were indicated in the figures.

### Reporting summary

Further information on research design is available in the Nature Research Reporting Summary linked to this article.

## Supplementary information

Supplementary Information

Description of Additional Supplementary Files

Supplementary Data 1

Reporting Summary

## Data Availability

Source data (Supplementary Data 1) are provided along with the paper. Materials related to the fusion protein expression are available upon reasonable request to lead contact Ming Zhou (mzhou@bcm.edu).
