# Supplementary material for: Transmembrane helices mediate the formation of a stable ternary complex of b5R, cyt b5, and SCD1
Source: Commun Biol. 2022 Sep 12;5:956. doi: 10.1038/s42003-022-03882-z (PMC9468158; doi:10.1038/s42003-022-03882-z)
Supplement: Supplementary file 1 — Supplementary Information [file 42003_2022_3882_MOESM1_ESM.pdf]

Supplementary Information

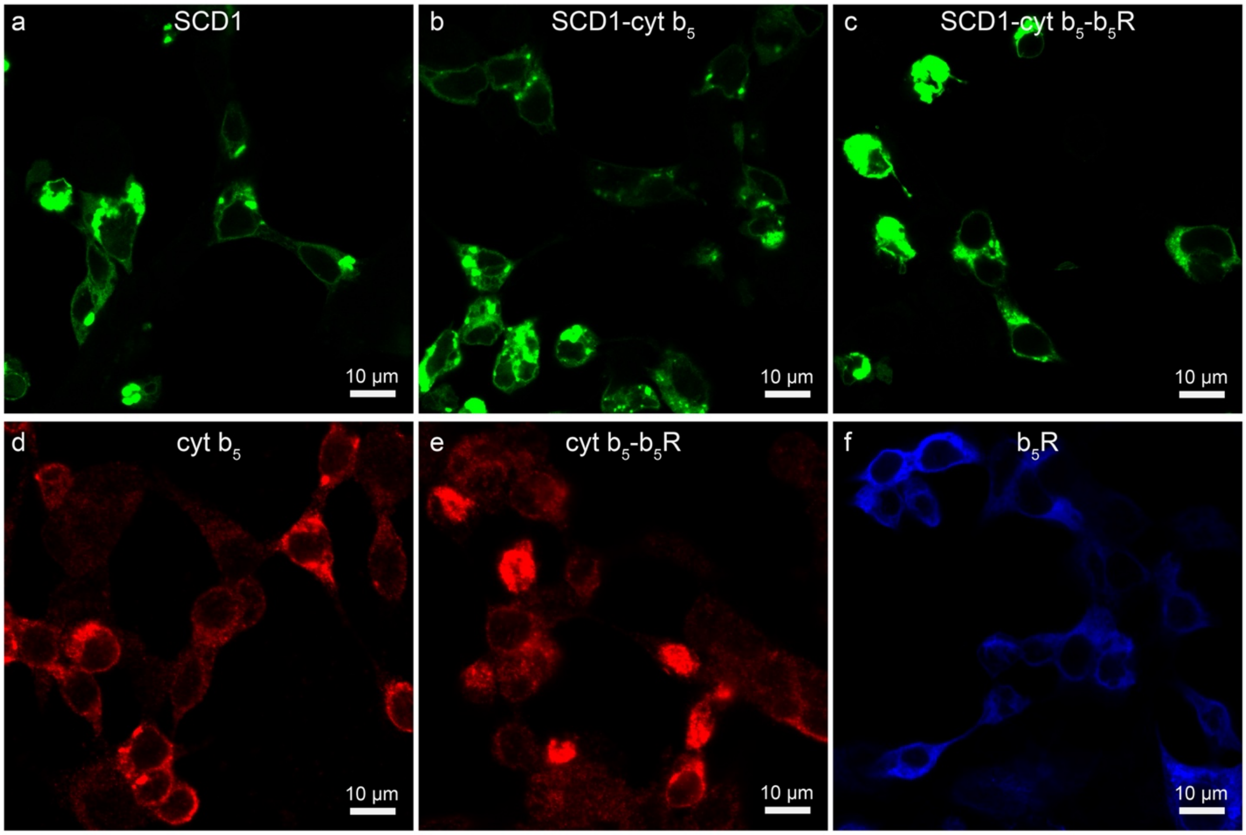

Supplementary Figure 1. Expression and subcellular distribution of SCD1, cyt b_5, b_5R, and their binary and ternary fusions. Confocal microscopy images of HEK293 cells expressing **a**, SCD1-GFP; **b**, SCD1-cyt b_5-GFP; **c**, GFP-SCD1-cyt b_5-b_5R; **d**, Myc-cyt b_5; **e**, Myc-cyt b_5-b_5R; and **f**, HA-b_5R.

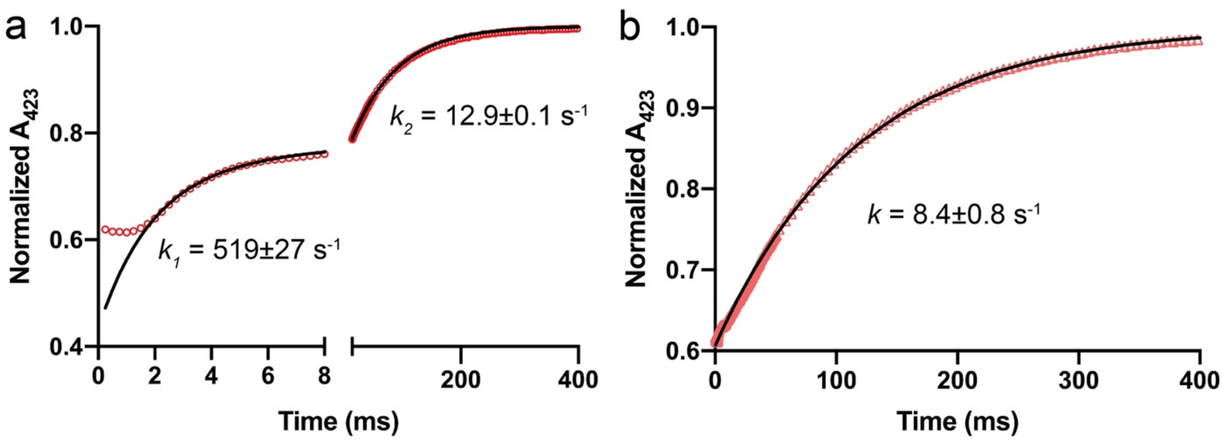

Supplementary Figure 2. Representative time courses of electron transfer between b_5R and cyt b_5 in **a**, cyt b_5-b_5R complex; and **b**, individual cyt b_5 and b_5R. In both **a** and **b**, the reaction buffer containing 150 mM NaCl was used. The reduction of cyt b_5 was followed at 423 nm. The time course is biphasic for cyt b_5-b_5R complex with a fast phase (k_1) and a slow phase (k_2), while monophasic (k) for the individual cyt b_5 and b_5R. Red dots represent experimental data and black curves the 1-exponential fit. Rate constants are denoted as mean ± SEM of three independent repeats.

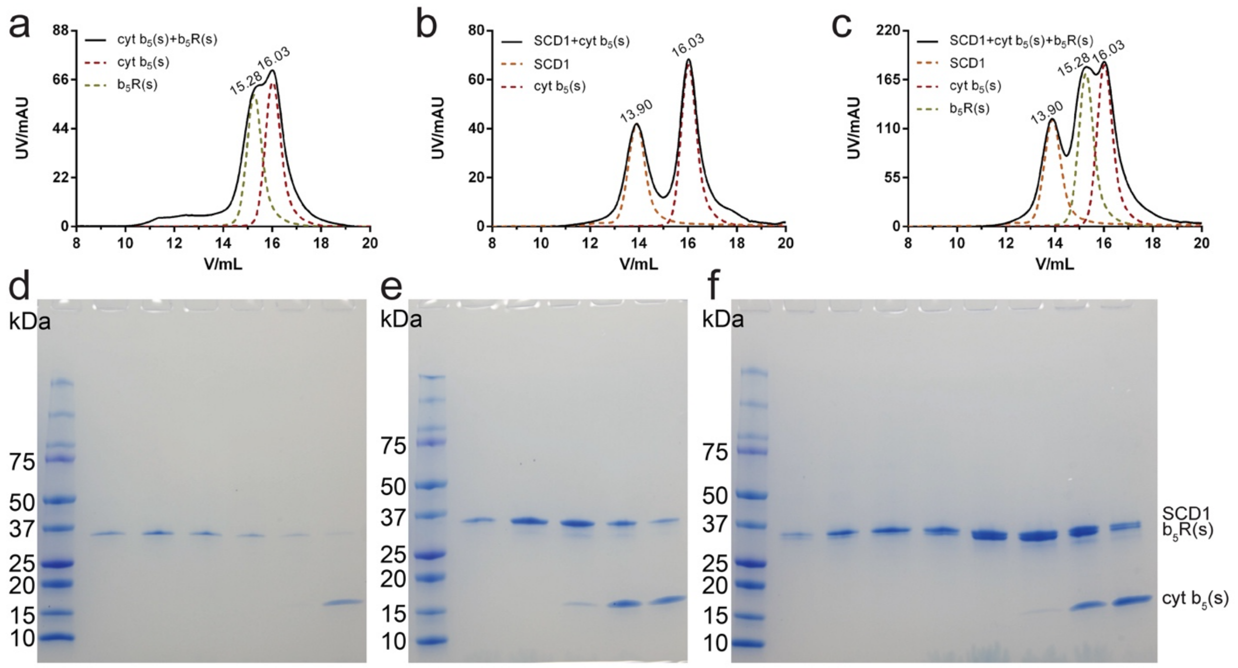

Supplementary Figure 3. SCD1, soluble cyt b_5, and b_5R cannot form a stable complex in SEC. **a** and **d**, SEC profile of a mixture of soluble cyt b_5 and b_5R, and SDS-PAGE gel image of fractions within the elution volume of 14 – 17 mL. **b** and **e**, SEC profile of a mixture of SCD1 and soluble cyt b_5, and SDS-PAGE gel image of fractions within the elution volume of 14 – 17 mL. **c** and **f**, SEC profile of a mixture of SCD1, soluble cyt b_5 and b_5R, and SDS-PAGE gel image of fractions within the elution volume of 13 – 17 mL.

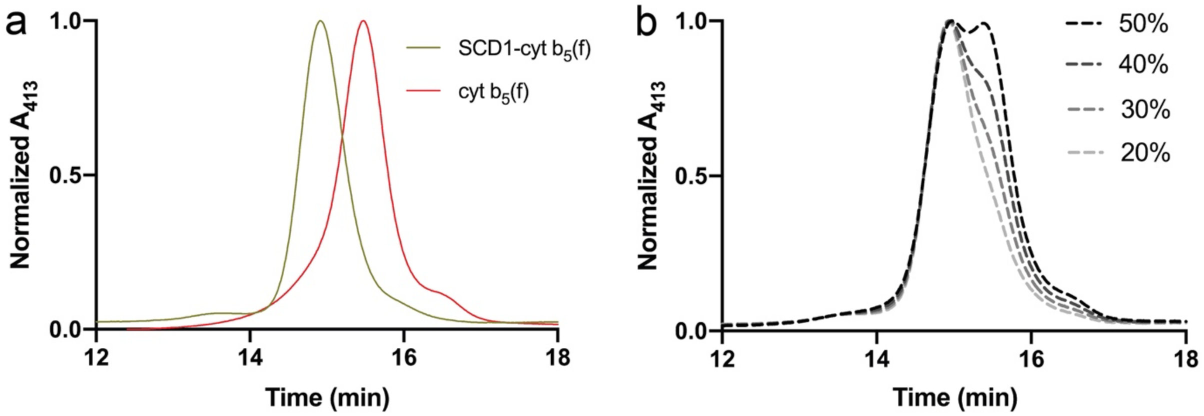

Supplementary Figure 4. a, SEC profiles of SCD1-cyt b_5 (yellow) complex and full-length cyt b_5 (red) in an HPLC system monitoring absorbance at 413 nm. **b**, Simulated profiles of a partially dissociated complex based on profiles in **a**. Percentages represent the molar ratio of dissociated cyt b_5 to all heme-containing proteins, including the SCD1-cyt b_5 complex and individual cyt b_5.

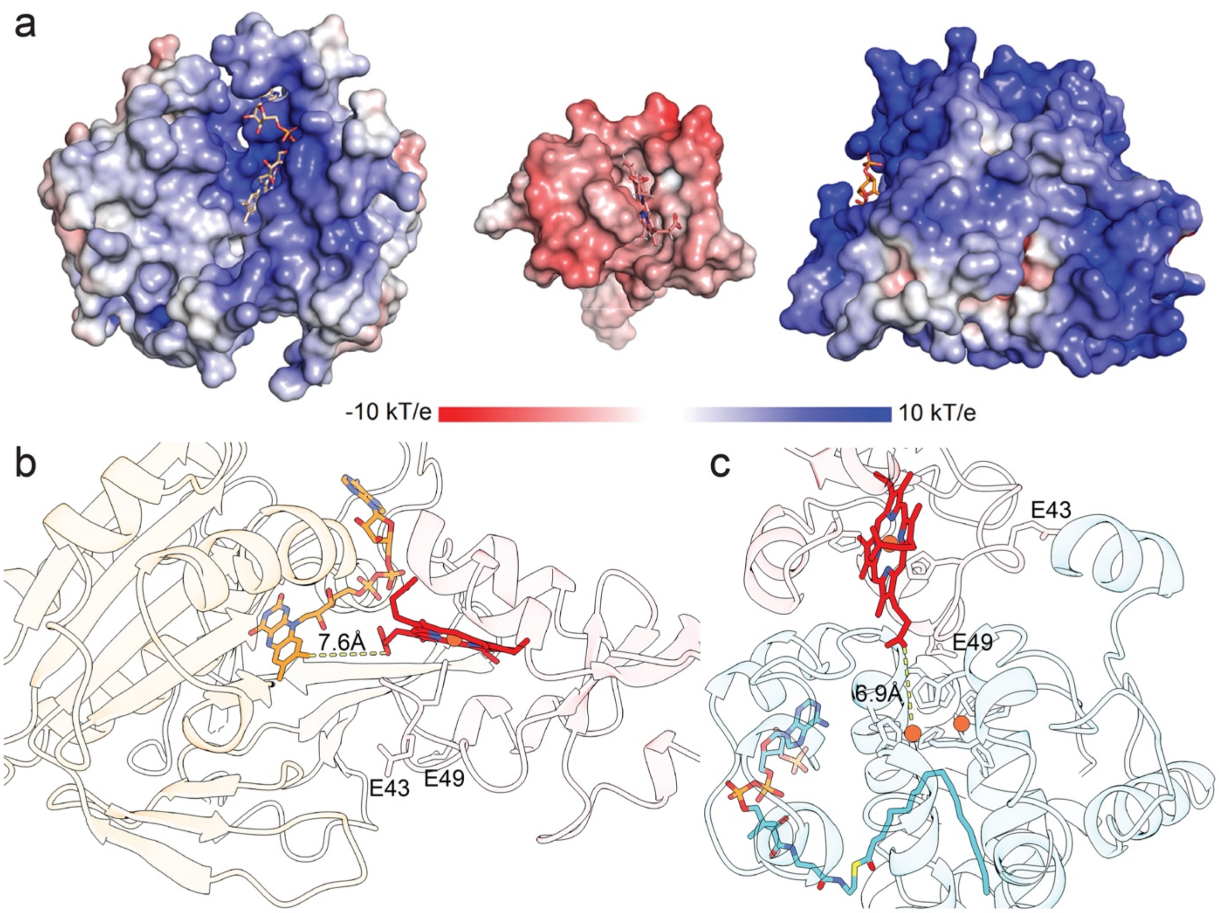

Supplementary Figure 5. a, Electrostatic potential of the soluble domains of b_5R (left), cyt b_5 (middle), and SCD1 (right). The binding interfaces are shown as surfaces colored from red to blue to represent potential from −10 kT/e to +10 kT/e. The prosthetic groups and ligands are shown as sticks. The docking model of: **b**, the soluble domains of cyt b_5 (red) and b_5R (orange); **c**, SCD1 (cyan) and the soluble domain cyt b_5 (red). The FAD in b_5R, heme in cyt b_5, and diiron center and acyl-CoA in SCD1 are highlighted. The closest distances between redox centers in the models are labeled. Two residues (E43 and E49) of cyt b_5 on the binding interfaces in both cyt b_5-b_5R and SCD1-cyt b_5 complex are shown as sidechain sticks.

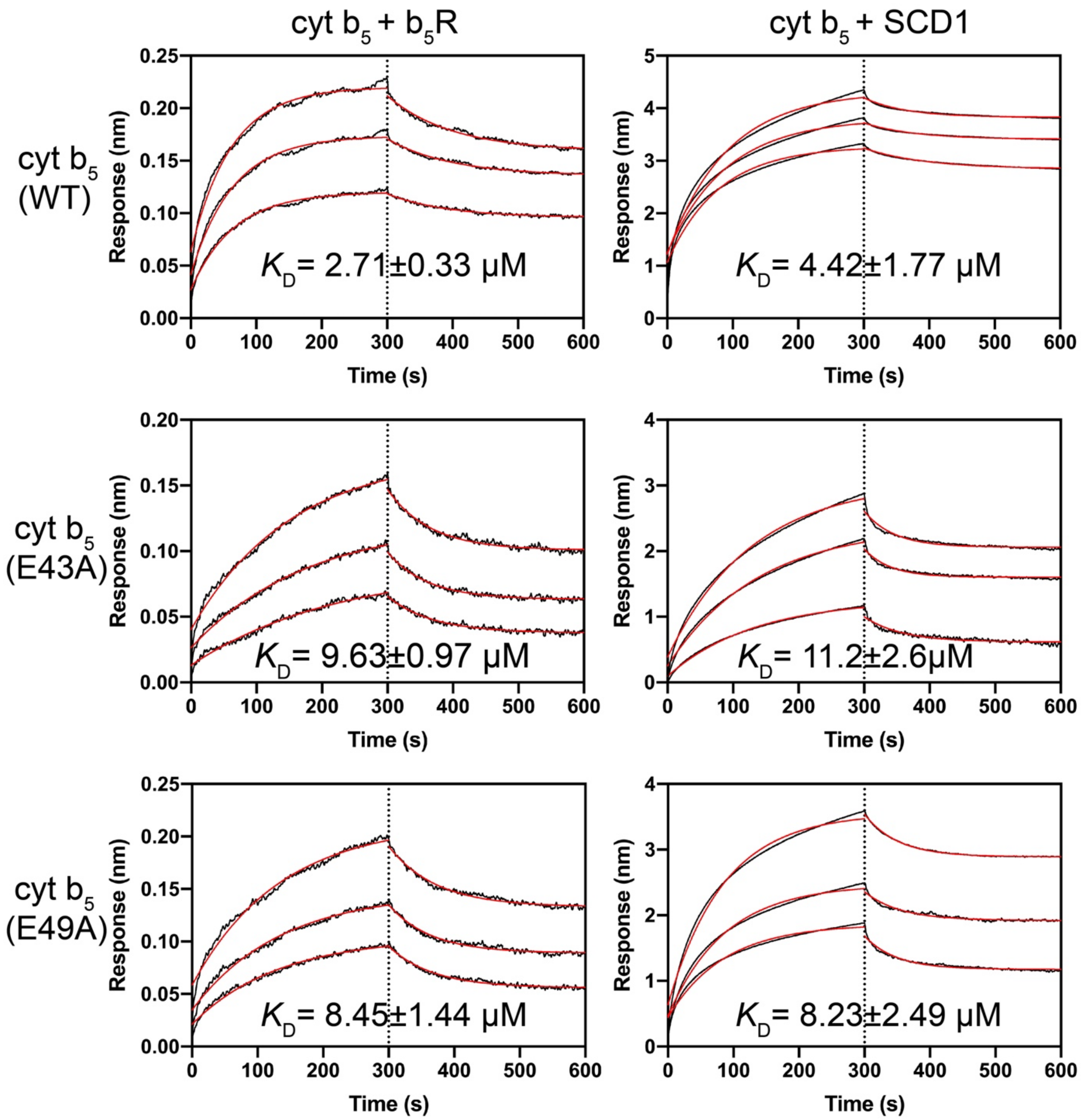

Supplementary Figure 6. Binding of wild-type (WT) and surface mutants (E43A and E49A) of cyt b_5 to b_5R (left panel) or SCD1 (right panel) measured in Octet BLI. K_D values were calculated as described in Methods.

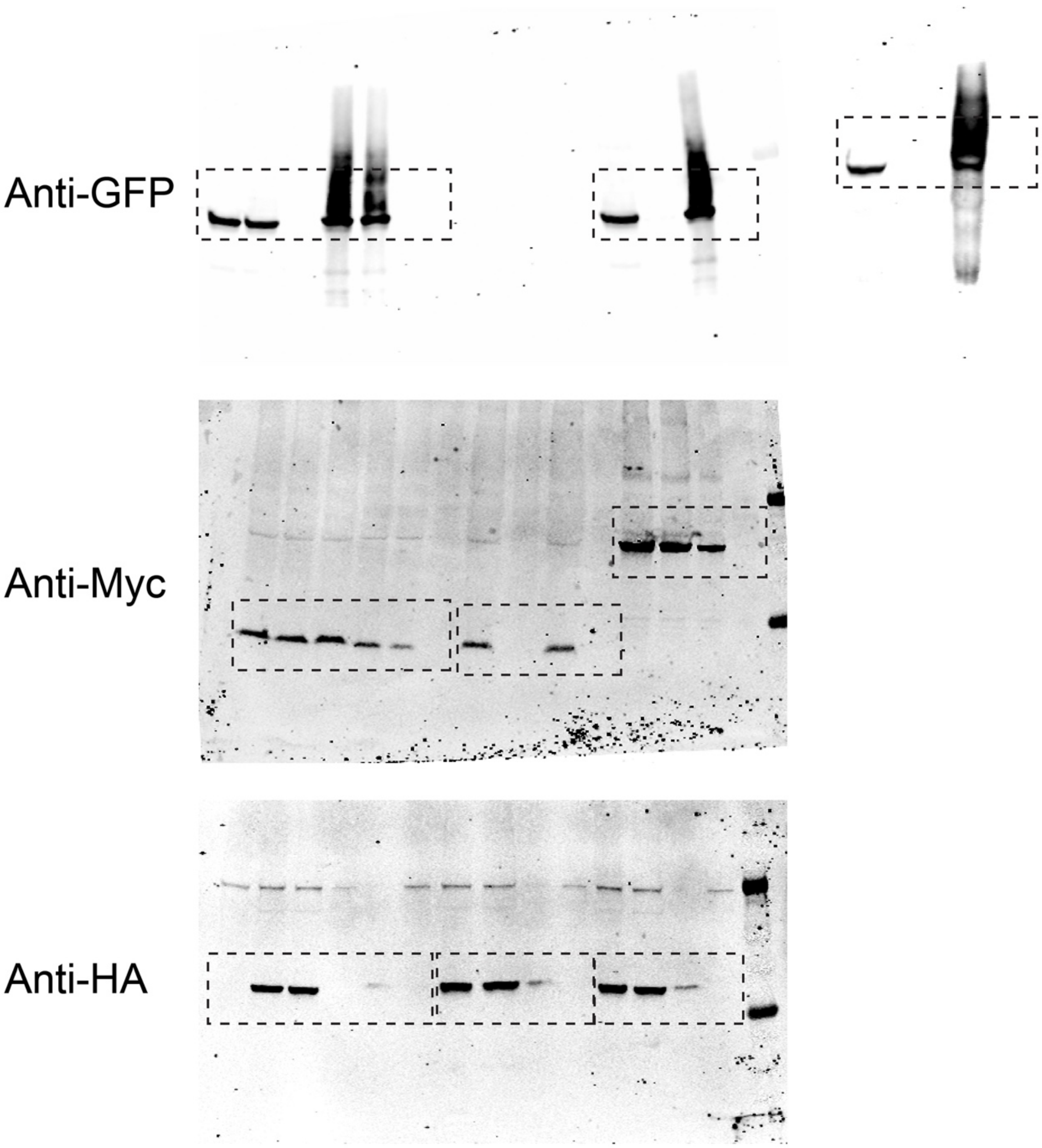

Supplementary Figure 7. Western blot images shown in **Figure 2**. Black dotted rectangles indicate regions of interest.
