# Supplementary material for: Transmembrane helices mediate the formation of a stable ternary complex of b5R, cyt b5, and SCD1
Source: Commun Biol. 2022 Sep 12;5:956. doi: 10.1038/s42003-022-03882-z (PMC9468158; doi:10.1038/s42003-022-03882-z)
Supplement: Supplementary file 2 — Description of Additional Supplementary Files [file 42003_2022_3882_MOESM2_ESM.pdf]

# Description of Additional Supplementary Files

**File name:** Supplementary Data 1
**Description:** The source data behind the Figure 3–5 in the paper.
