# Supplementary material for: Transmembrane helices mediate the formation of a stable ternary complex of b5R, cyt b5, and SCD1
Source: Commun Biol. 2022 Sep 12;5:956. doi: 10.1038/s42003-022-03882-z (PMC9468158; doi:10.1038/s42003-022-03882-z)
Supplement: Supplementary file 4 — Reporting Summary [file 42003_2022_3882_MOESM4_ESM.pdf]

nature research

Corresponding author(s): Ming Zhou

Last updated by author(s): Aug 5, 2022

Reporting Summary

Nature Research wishes to improve the reproducibility of the work that we publish. This form provides structure for consistency and transparency in reporting. For further information on Nature Research policies, see our Editorial Policies and the Editorial Policy Checklist.

Statistics

For all statistical analyses, confirm that the following items are present in the figure legend, table legend, main text, or Methods section.

n/a	Confirmed	
☐	☒	The exact sample size (n) for each experimental group/condition, given as a discrete number and unit of measurement
☐	☒	A statement on whether measurements were taken from distinct samples or whether the same sample was measured repeatedly
☐	☒	The statistical test(s) used AND whether they are one- or two-sided<br>*Only common tests should be described solely by name; describe more complex techniques in the Methods section.*
☒	☐	A description of all covariates tested
☒	☐	A description of any assumptions or corrections, such as tests of normality and adjustment for multiple comparisons
☐	☒	A full description of the statistical parameters including central tendency (e.g. means) or other basic estimates (e.g. regression coefficient) AND variation (e.g. standard deviation) or associated estimates of uncertainty (e.g. confidence intervals)
☒	☐	For null hypothesis testing, the test statistic (e.g. F, t, r) with confidence intervals, effect sizes, degrees of freedom and P value noted<br>*Give P values as exact values whenever suitable.*
☒	☐	For Bayesian analysis, information on the choice of priors and Markov chain Monte Carlo settings
☒	☐	For hierarchical and complex designs, identification of the appropriate level for tests and full reporting of outcomes
☒	☐	Estimates of effect sizes (e.g. Cohen's d, Pearson's r), indicating how they were calculated

Our web collection on statistics for biologists contains articles on many of the points above.

Software and code

Policy information about availability of computer code

Data collection	ZEN 2.5; UV-Vis ChemStation; LabSolution version 5.52; Octet Data Acquisition 12.0.1.8
Data analysis	ImageJ 1.53a; Prism 8.2.1; Pymol 2.5.2; ChimeraX 1.3; Postrun analysis (LabSolution version 5.52); Octet Data Analysis 12.0.1.8

For manuscripts utilizing custom algorithms or software that are central to the research but not yet described in published literature, software must be made available to editors and reviewers. We strongly encourage code deposition in a community repository (e.g. GitHub). See the Nature Research guidelines for submitting code & software for further information.

Data

Policy information about availability of data

All manuscripts must include a data availability statement. This statement should provide the following information, where applicable:

- Accession codes, unique identifiers, or web links for publicly available datasets
- A list of figures that have associated raw data
- A description of any restrictions on data availability

Source data are provided along with the paper. Any additional information required to reanalyze the data reported in this paper is available upon request to lead contact Ming Zhou (mzhou@bcm.edu).

Field-specific reporting

Please select the one below that is the best fit for your research. If you are not sure, read the appropriate sections before making your selection.

☒ Life sciences ☐ Behavioural & social sciences ☐ Ecological, evolutionary & environmental sciences

For a reference copy of the document with all sections, see nature.com/documents/nr-reporting-summary-flat.pdf

Life sciences study design

All studies must disclose on these points even when the disclosure is negative.

Sample size	No statistical method was used to determine sample size.
Data exclusions	No data were excluded from analyses.
Replication	All experiments were performed in triplicate or duplicate repeats. The number of biological repeats and statistical analyses for individual experiments are described in Methods or figure legends.
Randomization	Randomization is not relevant to this study.
Blinding	Blinding is not relevant to this study.

Reporting for specific materials, systems and methods

We require information from authors about some types of materials, experimental systems and methods used in many studies. Here, indicate whether each material, system or method listed is relevant to your study. If you are not sure if a list item applies to your research, read the appropriate section before selecting a response.

Materials & experimental systems

n/a	Involved in the study	
☐	☒	Antibodies
☐	☒	Eukaryotic cell lines
☒	☐	Palaeontology and archaeology
☒	☐	Animals and other organisms
☒	☐	Human research participants
☒	☐	Clinical data
☒	☐	Dual use research of concern

Methods

n/a	Involved in the study	
☒	☐	ChIP-seq
☒	☐	Flow cytometry
☒	☐	MRI-based neuroimaging

Antibodies

Antibodies used	Rabbit anti-Myc (Santa Cruz), mouse anti-HA (Santa Cruz), Alexa Fluor 555 anti-rabbit (Invitrogen/Thermo Fisher), Alexa Fluor 647 anti-mouse (Invitrogen/Thermo Fisher), mouse anti-GFP (Invitrogen/Thermo Fisher), mouse anti-Myc (Santa Cruz), and IRDye 800CW donkey anti-mouse (Licor)
Validation	All antibodies were validated in other publications listed at the source of suppliers.

Eukaryotic cell lines

Policy information about cell lines

Cell line source(s)	High Five cells (Invitrogen), HEK293F cells
Authentication	No further authentication was performed for commercially available cell lines.
Mycoplasma contamination	The cell lines were not tested for mycoplasma contamination.
Commonly misidentified lines (See ICLAC register)	No commonly misidentified lines were used in the study.
